# The NLRP3 Inflammasome May Contribute to Pathologic Neovascularization in the Advanced Stages of Diabetic Retinopathy

**DOI:** 10.1038/s41598-018-21198-z

**Published:** 2018-02-12

**Authors:** Shyam S. Chaurasia, Rayne R. Lim, Bhav H. Parikh, Yeo Sia Wey, Bo Bo Tun, Tien Yin Wong, Chi D. Luu, Rupesh Agrawal, Arkasubhra Ghosh, Alessandra Mortellaro, Elizabeth Rackoczy, Rajiv R. Mohan, Veluchamy A. Barathi

**Affiliations:** 1 Ocular Immunology and Angiogenesis Lab, Department of Veterinary Medicine & Surgery, University of Missouri, Columbia, MO USA; 2 Department of Biomedical Sciences, University of Missouri, Columbia, MO USA; 3 Ophthalmology, Harry S. Truman Memorial Veterans’ Hospital, Columbia, MO USA; 4 Translational Pre-Clinical Model Platform, Singapore Eye Research Institute, Singapore, Singapore; 5 The Ophthalmology & Visual Sciences Academic Clinical Program, DUKE-NUS Graduate Medical School, Singapore, Singapore; 6 Centre for Eye Research Australia, Department of Surgery (Ophthalmology), University of Melbourne, Melbourne, Australia; 7 National Healthcare Group Eye Institute, Tan Tock Seng Hospital, Singapore, Singapore; 8 GROW laboratory, Narayana Nethralaya, Bangalore, India; 9 Singapore Immunology Network (SIgN), Agency for Science, Technology and Research (A*STAR), Singapore, Singapore; 10 Centre for Ophthalmology and Visual Sciences, University of Western Australia, Perth, WA 6009 Australia; 11 Mason Eye Institute, University of Missouri, Columbia, MO USA

## Abstract

Diabetic retinopathy (DR) is a retinal microvascular disease characterized by inflammatory and angiogenic pathways. In this study, we evaluated NLRP3 inflammasome in a double transgenic mouse model, Akimba (*Ins2*^*Akita*^*xVEGF*^+*/*−^), which demonstrates hyperglycemia, vascular hyperpermeability and neovascularization seen in the proliferative DR. Retinal structural integrity, vascular leakage and function were examined by fundus photography, fluorescein angiography, optical coherence tomography, retinal flat mounts, laser speckle flowgraphy (LSFG), and electroretinography in Akimba and its parental strains, Akita (*Ins2*^*Akita*^) and Kimba (*trVEGF029*) mice. Inflammatory mechanisms involving NLRP3 inflammasome were investigated using real time-PCR, immunohistochemistry, ELISA and western blots. We observed an increased vascular leakage, reduced retinal thickness, and function in Akimba retina. Also, Akimba retina depicts decreased relative flow volume measured by LSFG. Most importantly, high levels of IL-1β along with increased NLRP3, ASC, and Caspase-1 at mRNA and protein levels were observed in Akimba retina. However, the *in vivo* functional role remains undefined. In conclusion, increased activation of macroglia (GFAP), microglia (Iba-1 and OX-42) and perivascular macrophages (F4/80 and CD14) together with pro-inflammatory (IL-1β and IL-6) and pro-angiogenic markers (PECAM-1, ICAM-1, VEGF, Flt-1, and Flk-1), suggested a critical role for NLRP3 inflammasome in the Akimba mouse model depicting advanced stages of DR pathogenesis.

## Introduction

Diabetic retinopathy (DR) is a common retinal microvascular complication of diabetes mellitus in working adults^1,2^. Clinically, DR is broadly categorized into an early stage non-proliferative DR (NPDR) and the advanced stage of proliferative-DR (PDR), with and without the development of diabetic macular edema (DME). DR involves multiple interlinked pathways that lead to cellular damage, vascular and neuronal abnormalities, and microvascular damage in the retina^3,4^. Chronic low-grade retinal inflammation caused by the activated microglia/macrophages have been described as a major contributor in the pathogenesis of DR and PDR responsible for the infiltration of leukocytes into the retina, release of inflammatory mediators, breakdown of the blood-retinal barrier (BRB), vascular leakage and neovascularisation^5–7^. In clinical studies, elevated serum and vitreous levels of pro-inflammatory cytokines such as C-reactive protein (CRP), Interleukin-1beta (IL-1β), Interleukin-6 (IL-6) and Tumour Necrosis Factor-alpha (TNF-α) have been demonstrated in DR and PDR^8,9^.

The immune system in the retina is evolutionally exempted from deleterious effects of exogenously triggered pathogen-associated molecular patterns (PAMPs), as well as endogenously generated damage-associated molecular patterns (DAMPs), sometimes referred to as alarmins^10^. These DAMPs are recognized by pattern recognition receptors (PRRs) such as toll-like receptors (TLRs), C-type lectin receptors (CLR), RIG-like receptors (RLR), and NOD-like receptors (NLR)^11,12^. Growing evidence suggest the involvement of DAMPs-bound PRRs in the induction of low-grade but chronic inflammatory responses seen in many ocular and non-ocular multifactorial diseases such as age-related macular degeneration, Alzheimer’s disease, Parkinson’s disease and multiple sclerosis^13–15^. The DAMPs such as oxidized lipoproteins, glycated proteins, uric acid, DNA and RNA from necrotic cells have been suggested to contribute to the pathogenesis of retinal diseases^16,17^. This may compromise the first line of defense system embodied by the innate immune system and protected by the blood-retina barrier (BRB) as seen in PDR and DME^18^.

DAMPs help to assemble a newly identified multimeric protein complex called inflammasome comprised of NLRP3 (NLR family pyrin domain containing 3), apoptosis associated speck-like protein (ASC) and Caspase-1^19^. The NLRP3 component recognizes danger signals and assembles the protein complex ASC to activate caspase-1, which leads to the proteolytic secretion of the pro-inflammatory cytokine, IL-1β. A recent study on metabolic disorders such as obesity and type 2 diabetes have also shown to involve activated NLRP3 inflammsome^20^. Also, mice deficient in NLRP3 components showed attenuated diabetic phenotype^21^. Moreover, a recent report found traces of NLRP3 expression in the vitreous of PDR patients^22^. Although inflammatory pathways play a substantial role in DR pathogenesis, especially in the proliferative and angiogenic stages, the contribution of the NLRP3 inflammasome has not been described.

One of the major limitations to study late stage DR pathogenesis is the unavailability of a suitable animal model. To date, several DR rodent models have been reported, but they mostly represent early stages of DR and do not depict the pathology seen in the advanced stages of DR (PDR and DME). This could be probably due to the short lifespan and duration of diabetes. Various animal models with the detailed description of pros and cons have been summarized in the literature^23,24^. The most common rodent model described is streptozotocin (STZ)-induced type-I diabetes, which develops early symptoms of DR as early as 2–3 months characterized by pericyte loss, thickening of basement membrane and increased vascular permeability. However, advanced proliferative and neovascular changes were not observed in these rodents eyes even after 18–24 months of diabetes^25,26^.

Recently, mouse models such as Akita (*Ins2*^*Akita*^) and Kimba (*trVEGF029*) have been described in DR studies. The Akita mouse is a spontaneous type-I diabetes model that carries a dominant point mutation in the insulin-2 gene^27^. It develops hyperglycemia and exhibits early pathophysiological changes of DR but fails to progress to the advanced stage. The Kimba mouse, which has transient photoreceptor (PhR)-specific overexpression of human VEGF_165_ (hVEGF), depicts a less destructive and slower form of retinal neovascularization present in the advanced stages of DR^28^. Although this model has several vascular abnormalities seen in DR, it lacks hyperglycemic background. Due to the limitations of these mouse models, Akimba (*Ins2*^*Akita*^*xVEGF*^+*/*−^) mice were generated by crossing Kimba and Akita^29^ described with advanced retinal lesions, increased leaky capillaries, venous beading, tortuous vessels and attenuation of vessels, characteristic features seen in human DR, providing a significant advancement in DR research^24,30^. Since inflammatory pathways contribute significantly to DR, we designed this study to evaluate the role of the NLRP3 inflammasome in Akimba retina to better understand the disease pathogenesis in the advanced stages of DR.

## Results

### Akimba mouse displays characteristic features of advanced stages of DR

**Figure 1:**
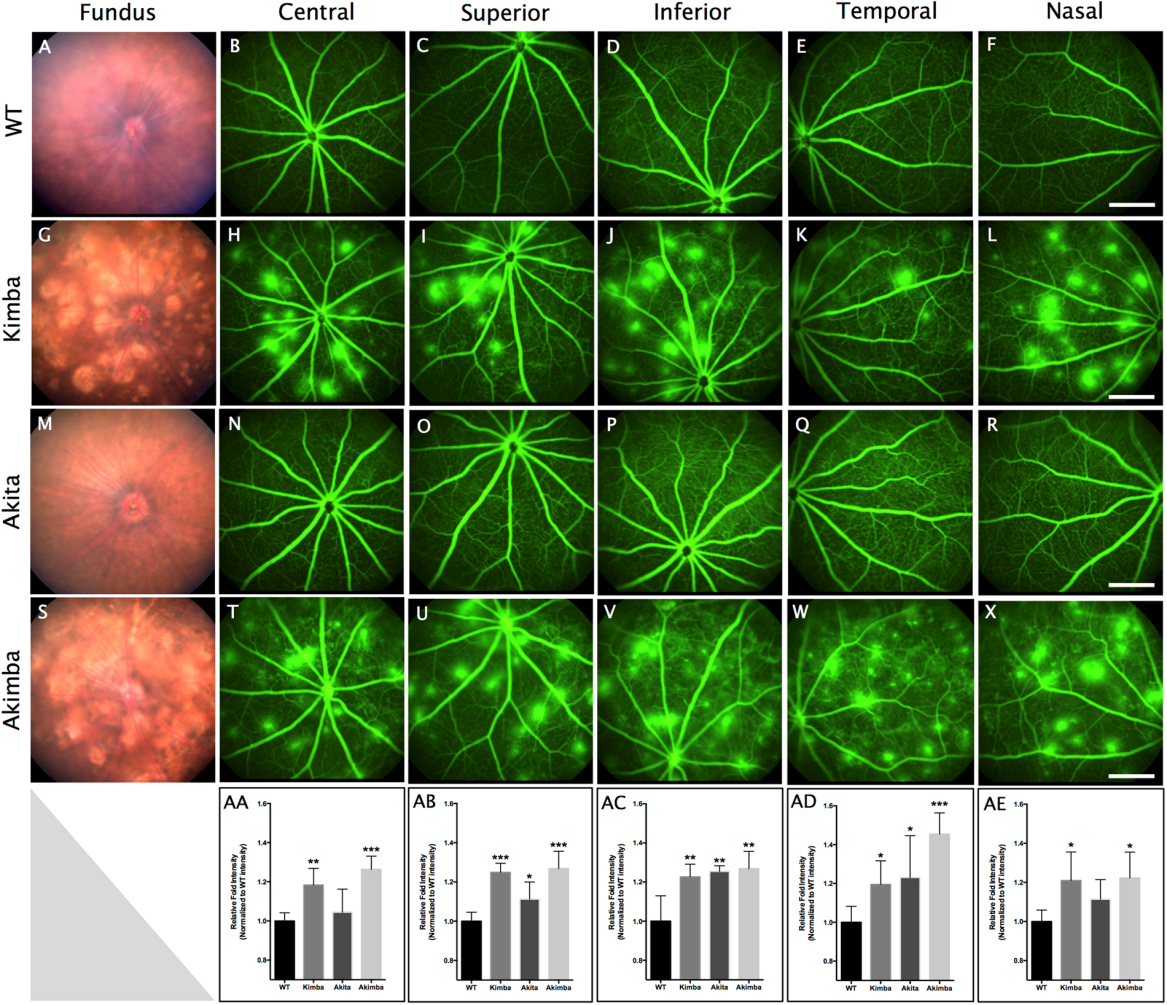
Color fundus photographs and Fundus fluorescein angiograms (FFA) of wild -type (WT), Kimba, Akita and Akimba mice. WT mouse showed regular arterial and venular caliber, branching, and tortuosity in the retina (**A**–**F**). Kimba mice had multiple spots of hyperfluorescein (bright spots) throughout the retina (**G**–**L**). Akita mice displayed a slight increase in tortuosity with no signs of leakage (**M–R**). Double transgenic Akimba mice showed vasculature abnormalities inherited from parental strains, with high severity of leakages accompanied with tortuosity and significant loss of capillary density (**S**–**X**). Relative fluorescein intensity calculated using mean grey area measurement of FFA images showed a significant increase in fluorescein leakage in Akimba mice across all photographed areas of the retina (**AA–AE**). n = 8 animals in each group. *p < 0.05; **p < 0.01 and ***p < 0.001 compared to WT group. Data represent mean ± SD. Scale bar, 500 µm.

The fundus photographs and fluorescein angiograms from the Akimba mouse display increased retinal vascular leakage and vascular changes observed in DR compared to their parental strains. In the present study, we describe central, superior, inferior, temporal and nasal retina photographs in 20–24 weeks old mouse retina to further describe the abnormalities in the retinal microvascular (Fig. 1A–X). The Akimba mouse (Fig. 1S–X) was characterized by capillary dropout, severe hemorrhage, neovascularization, venous beading and vessel tortuosity compared to the WT or parental strains (Fig. 1A–F). Kimba mouse showed less severe but similar vascular phenotype (Fig. 1G–L). Akita showed no leaky vessels and relatively mild phenotype compared to the other animal models studied (Fig. 1M–R). We measured relative fluorescein intensity using a mean grey area measurement of FFA images and found a significant increase in fluorescein leakage in Akimba mice across all areas of the retina (Fig. 1 AA–AE).

### Akimba mouse has neurodegenerative retina and aberrant retinal function

**Figure 2:**
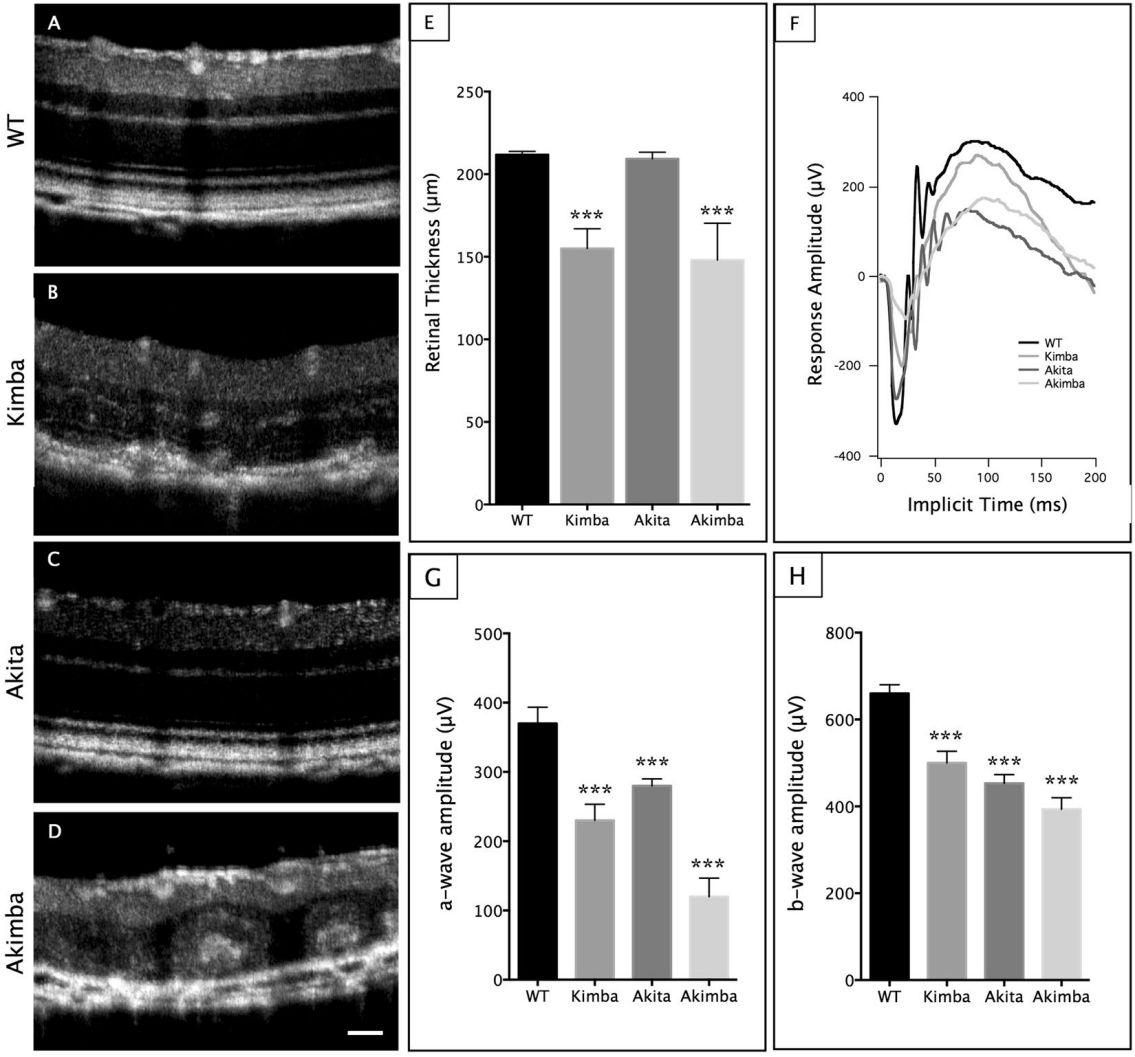
Akimba retina displayed reduced retinal thickness and disrupted retinal function. Optical coherence tomography (OCT) analysis showed disruption of retinal architecture and appearance of subretinal hemorrhage in (**C**) Kimba and (**D**) Akimba retina. This was accompanied by a significant reduction in retinal thickness in these mice (**E**). Abnormal ERG was observed in all the mice models (**F**–**H**), with significantly decreased scotopic a-wave (**G**) and b-wave (**H**) amplitudes. Akimba mice showed the most severe disruption of retinal function, corresponding with the loss of retinal thickness and subretinal hemorrhages. n = 6 animals in each group. ***p < 0.001 compared to WT. Data represent mean $\pm$ SD. Scale bar, 50 µm.

OCT imaging showed no significant difference in retinal thickness (Fig. 2E) between Akita (Fig. 2C) and WT (Fig. 2A) mice, but there was significant reduction in the retinal thickness in Akimba (Fig. 2D; p < 0.001) and Kimba mice (Fig. 2C; p < 0.001) compared to the WT controls. We next measured retinal function using ERG (Fig. 2F) and found the average scotopic a- and b-wave amplitudes (Fig. 2G, H, respectively) obtained at the flash intensity of 10 cd·s/m^2^ were significantly reduced in all the experimental groups studied compared to the WT (p < 0.001 for both a- and b-wave amplitude). Akimba showed the maximal decrease in the a- and b-wave amplitudes compared to their parental strains (Fig. 2F).

### Akimba retina shows decreased relative blood flow volume

**Figure 3:**
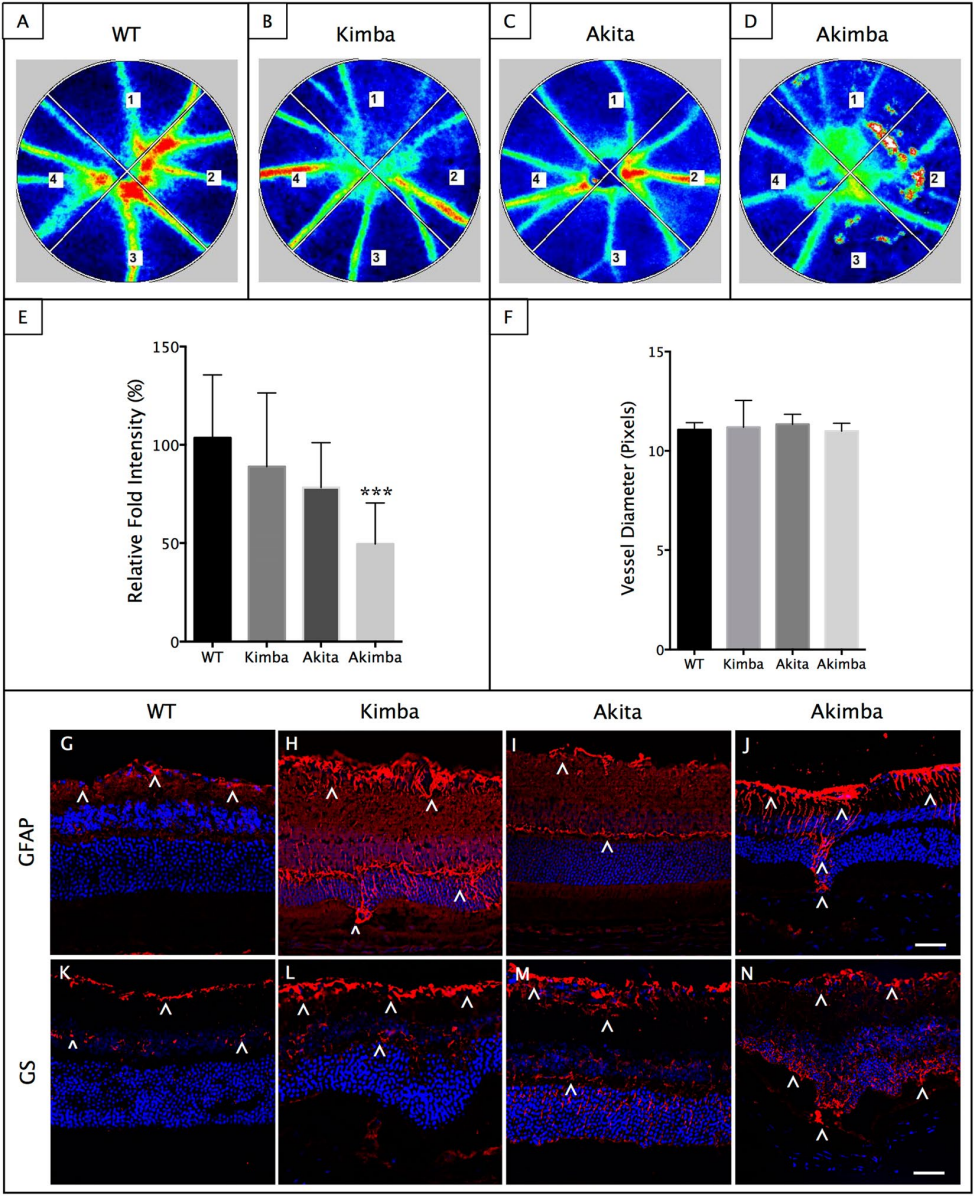
Reduced retinal flow volume and Müller cell gliosis in Akimba mouse. Laser speckle flowgraphy (LSFG) heat map of retinal blood flow in the animal models (**A**–**D**). Red indicates faster blood flow while blue indicates slower blood flow. Numbers 1–4 denotes four quadrants of the retina used for the analysis. Assessment of relative flow volume in the all four quadrants depicted significant attenuation of blood flow in Akimba (**D, E**). No differences were seen between the Kimba and Akita mice (**E**). Measurement of vessel diameter showed no difference in caliber amongst all four groups (**F**). Data represent mean ${\rm{\pm }}$ SD. n = 6 animals in each group. ***p < 0.001 compared to the WT. Akimba mouse showed maximal GFAP (**J**) stained cells compared with WT (**G**) or the parental strains (**H**,** I**). Similarly, GS immunostaining was observed throughout the entire inner retina and few cells migrating to the outer retina in the Akimba mice (**N**). Kimba retina showed similar staining patter but less intense (**L**) followed by Akita, where we found staining restricted to the inner retina (**M**) compared to the WT (**K**). n = 4 retina per group for immunohistochemistry, repeated twice. Arrowheads indicate positively stained cells. Scale bar, 50 µm.

Retinal glial cells are known to influence the activity-dependent regulation of blood flow in the pathogenesis of many ocular diseases, including DR^31^. Recently, a novel non-invasive method has been defined to calculate the relative blood flow volume (RFV) in the human retina using laser speckle flowgraphy (LSFG)^32^. RFV is influenced by vessel diameter and retinal flow velocity and derived by filtering the background choroidal flow from the overall blood flow value in a region of interest centered on a retinal vessel (Fig. 3A–D). Akimba retina showed a significant decrease in RFV compared to its parental strains (Fig. 3D, E, P < 0.001). However, there were no differences in the retinal vessel diameter measured among the animal models studied (Fig. 3F).

### Akimba retina exhibits hyperactive micro- and macroglial cells and increased mobilization of perivascular macrophages

PDR is primarily considered microangiopathy and neovascularization disease, but several reactive changes in Müller cells and activation of the resident or migrating macrophages/microglial cells have been reported in the disease pathogenesis^33^. We found a massive upregulation of GFAP stained macroglial cells throughout the Akimba retina (Fig. 3J) and relatively less expression in Kimba (Fig. 3H) followed by Akita (Fig. 3I) and further fewer cells in the inner retina of the WT (Fig. 3G) mice. A similar trend was observed with GS staining (Fig. 3K–N).

**Figure 4:**
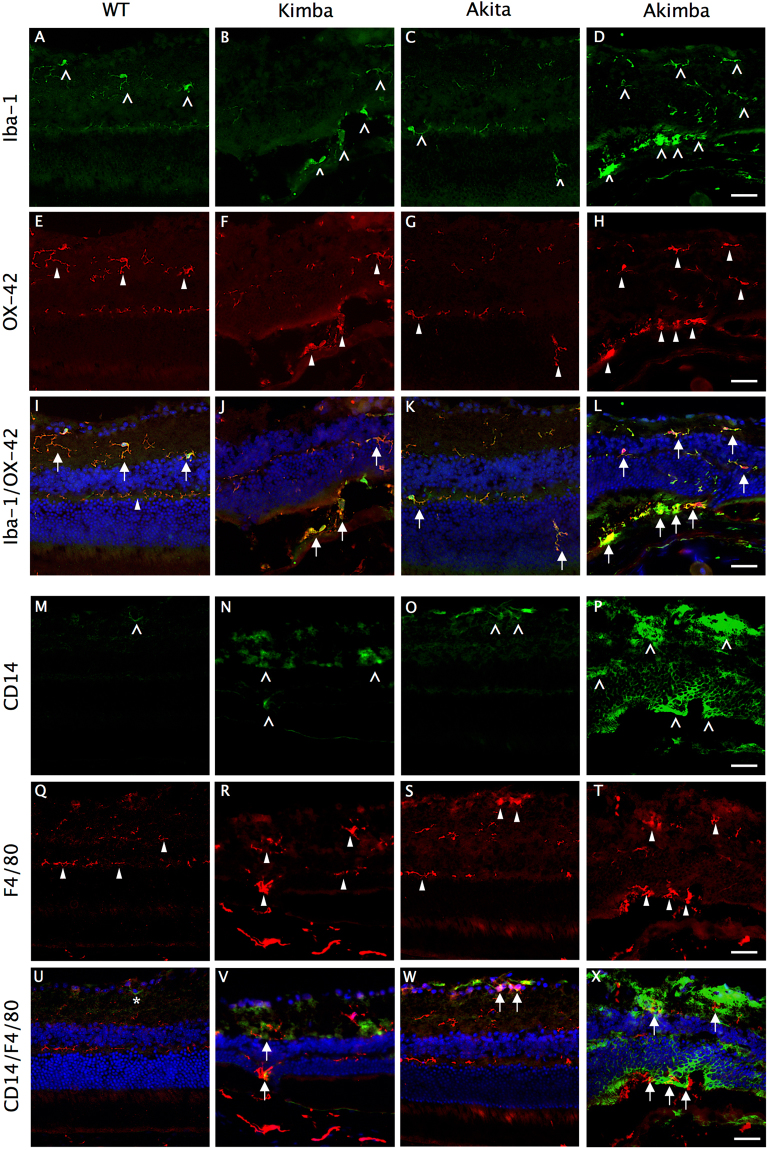
Elevated expression of macrophage/microglia markers in Akimba mouse. Double immunostaining of Iba-1 (**A**–**D**) with OX-42 (**E–H**), and CD14 (**M**–**P**) with F4/80 (**Q**–**T**). Iba-1 staining (arrowheads) in WT (**A**) mice showed resident microglial cells with normal ramified morphology in the inner retina. Akita (**C**) mice showed minimal activation of microglia, however, had displaced microglia in the outer nuclear layer (ONL). Iba-1+ cells were both increased and amoeboid shaped in the outer retina of Kimba (**B**), and maximal activation was seen in Akimba (**D**) retina. OX-42 (block arrowheads) stains a cell surface antigen expressed by microglia, hence co-localized (arrows) with Iba-1+ microglia in WT (**I**). Majority of Iba-1+ microglia in Kimba (**J**), Akita (**K**) and Akimba (**L**) mice retina also stained positive for OX-42 (arrows). CD14 (arrowheads) is expressed by perivascular macrophages, which were minimal and located only around the vessels in the ganglion cell layer of the WT (**M**) mice. Increased expression was seen in the Kimba (**N**) and Akita (**O**) mice, and was drastically elevated in the outer retina of Akimba (**P**) mice, indicating migratory perivascular cells around new vessel sprouting from choroid into photoreceptor (PhR) region. F4/80 (block arrowheads) staining for macrophage showed predominant localization in the inner retina of the WT (**Q**) mice, which increased in Kimba (**R**) and Akita (**S**) mice, and was significantly elevated in GCL and ONL of Akimba (**T**) mice. While naïve CD14+ cell was not positive for F4/80 in WT mice (**U**, asterisk), co-localization (arrows) was seen in the GCL of Akita (**W**) mice, and in ONL/PhR of the Kimba (**V**) and Akimba (**X**) mice retina where sprouting vessels were located. n = 4 retina per group; repeated twice. Scale bar, 50 µm.

The presence of activated microglial cells (Iba-1 and OX-42) and resident or infiltrating macrophages (F4/80 and CD14) were confirmed by double immunostaining. In the normal retina, several ramified Iba-1 (Fig. 4A) and OX-42 (Fig. 4E) positive cells were observed in the outer plexiform layer (OPL), ganglion cell layer (GCL) or clustered around the perivascular region in the retina. In Kimba (Fig. 4B, F) and Akimba (Fig. 4D, H), these cells appeared hypertrophic or amoeboid and scattered in the entire retina. The most remarkable observation was the infiltration of these cells into the outer retina in Akimba mouse (Fig. 4L). In contrast, Akita (Fig. 4C, G) showed a marginal increase in activated microglial cells but none infiltrating into the outer retina.

 The second category of microglial precursors, also called perivascular macrophages, appear around the blood vessels and upon activation gradually lose dendritic processes and transform typically into phagocytic macrophages. In fact, activated macrophages have been reported earlier in the proliferative vitreoretinal disorders^34^. In Akita, we observed scant staining of F4/80 in the OPL (Fig. 4S). In both Kimba (Fig. 4R) and Akimba (Fig. 4T), F4/80 positive cells were seen in the photoreceptor layers. In contrast, CD14 was primarily present in the perivascular zones around the vessels in the ganglion cell layer of the WT (Fig. 4M) and Akita mice (Fig. 4O). Increased expression was seen in the Kimba (Fig. 4N), which was highly elevated in the outer retina of Akimba mice (Fig. 4P), indicating migratory perivascular cells around new vessel sprouting from choroid into photoreceptor (PhR) region. We observed that only a few F4/80 positive cells were co-localized with CD14 stained cells in Akita and Kimba mice (Fig. 4W, V, respectively), which amplified immensely in in Akimba (Fig. 4X) and none in WT retina (Fig. 4U).

**Figure 5:**
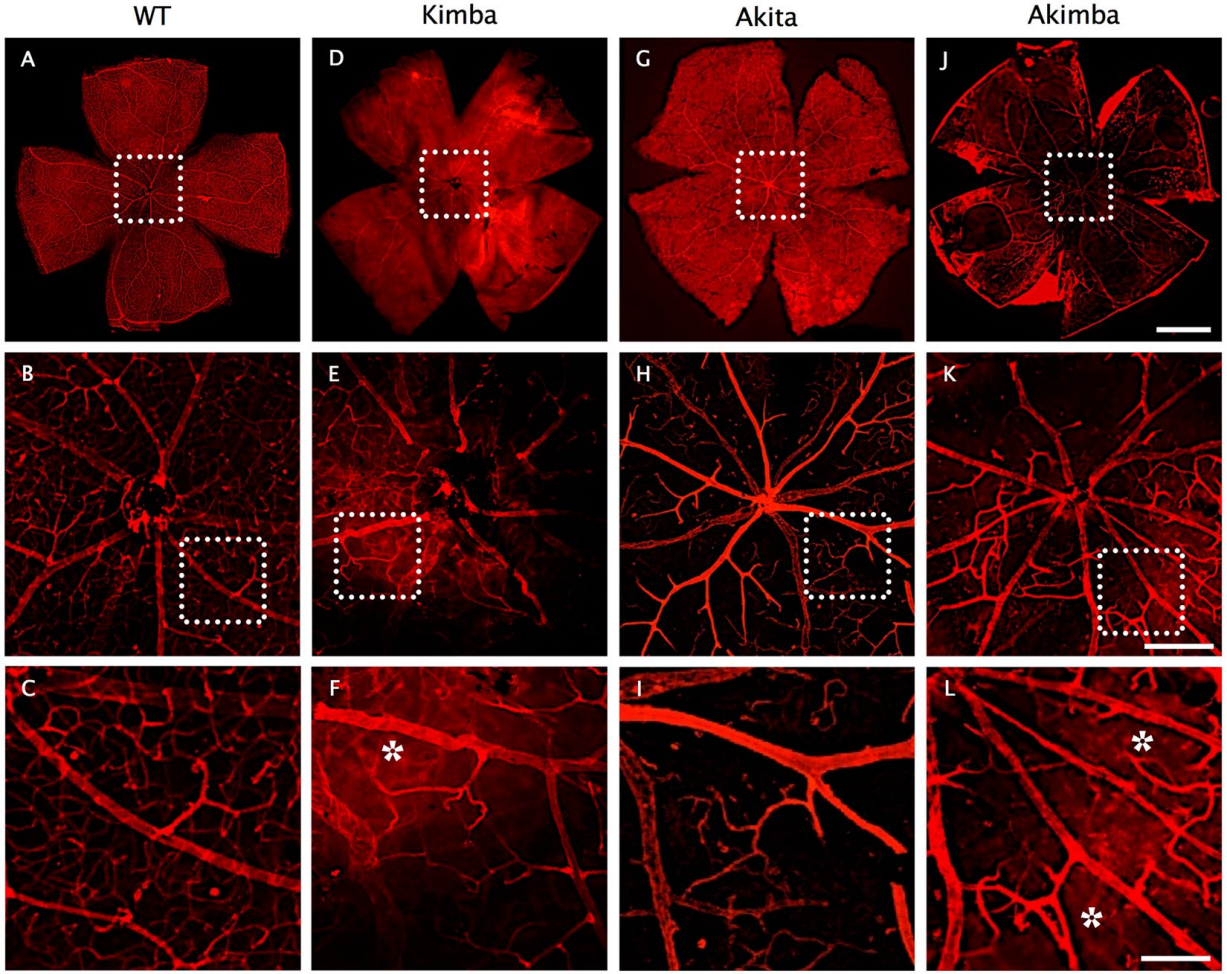
Loss of retinal capillary density and vessel leakage in Akimba mice. Isolectin-GS IB4 staining on retinal flat mount showed normal vascular bed and uniform capillary distribution in WT (**A**–**C**) mouse, which was reduced markedly in Kimba (**D**–**F**), Akita (**G**–**I**) and significantly higher in Akimba (**J**–**L**) mice. Disruption of the capillary network was noted especially around the optic disk of Akimba (**K**) retina. Diffused staining (asterisk) around the optic head of Kimba (**F**) and Akimba (**L**) mice indicate loss of vessel integrity and leakage. n = 4 retina per group. Each retina flatmount image (**A, D, G**, and **J**) was stitched from at least 8 images were taken at 50× magnification; scale bar, 1 mm. Insets shown in middle row (**B, E, H**, and **I**) were taken at 100×; scale bar, 200 µm. Insets are shown in bottom row (**C, F, I**, and **L**) were taken at 200×; scale bar, 100 µm.

Whole retina flat-mounts stained using Alexa Fluor 594-conjugated Griffonia simplicifolia (Bandeiraea) isolectin B4 indicate varying degrees of vascular permeability/leakage and altered retinal arteriolar microvasculature in all the animal models studied (Fig. 5D–L). BRBs were severely compromised in Akimba (Fig. 5J–L) retinas compared to Akita (Fig. 5G-I) and Kimba (Fig. 5D–F) as evidenced by the significantly increased capillary dropouts and severe vascular degeneration. Akimba mouse also exhibits retinal lesions accompanied with several instances of retinal neovascularization depicting advanced stages of DR (Fig. 5J–L).

### Akimba retina is characterized by increased IL-1β levels and high inflammatory index

**Figure 6:**
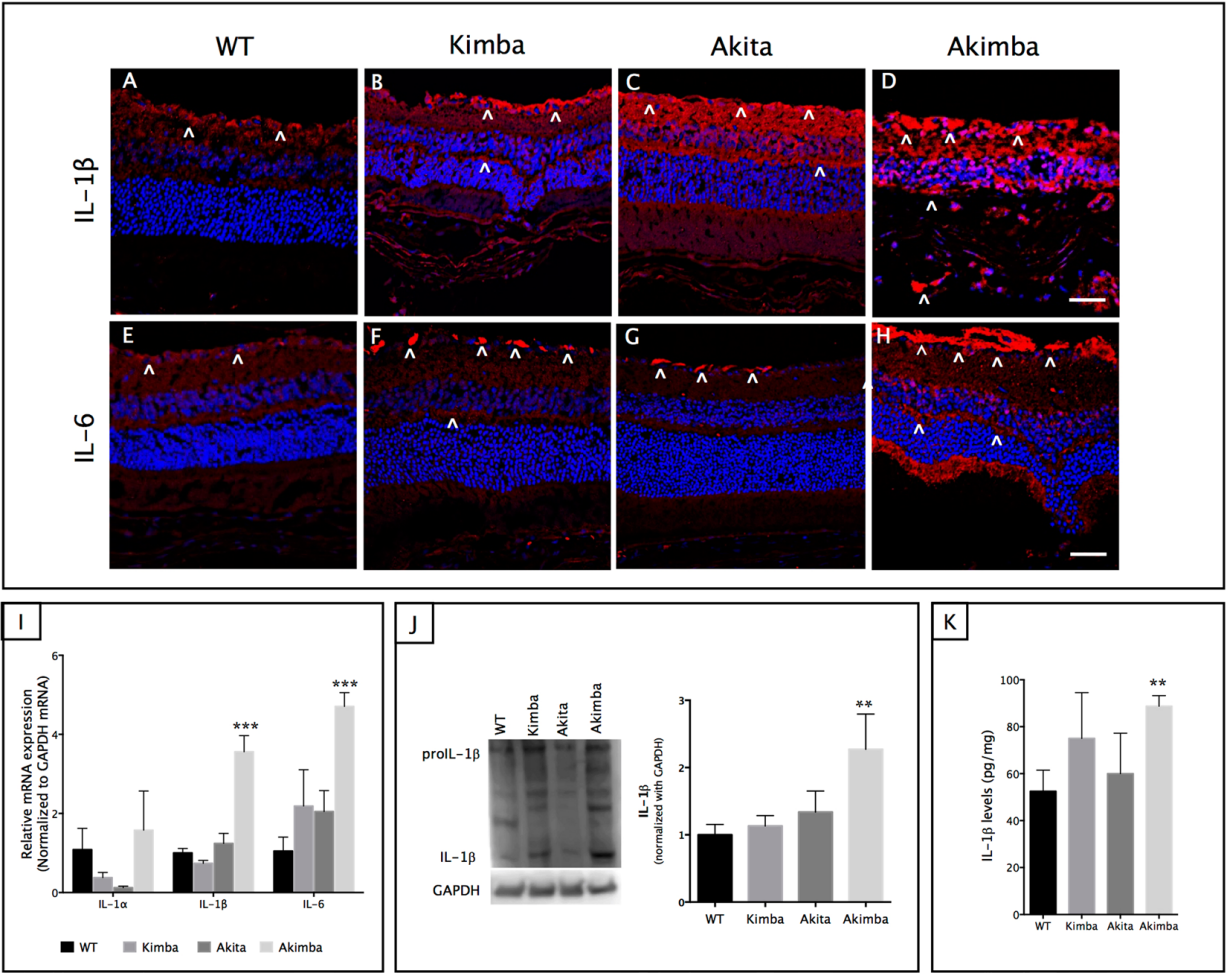
Akimba mice showed significantly high levels of the inflammatory milieu. Immunostaining with IL-1β (**A**–**D**) and IL-6 (**E–H**) showed basal levels of these cytokines in the WT (**A, E**, respectively), which were markedly increased in all the mice models studied. Akita showed diffused expression of IL-1β from the GCL to the OPL (**C**), which gradually increased in Kimba (**B**) followed by maximal expression in the Akimba (**D**) retina where we found its expression to be localized in the entire retina. We observed few specks of IL-6 staining in the GCL of WT (**E**) mice, with increasing expression levels from Akita (**G**), Kimba (**F**) with maximal expression in Akimba (**H**). Real-time PCR (**I**) followed the similar expression trend seen in immunostaining. The most remarkable findings were the significant high levels of IL-1β expression exclusively in Akimba retina found in western blots (**J**) and recapitulated with ELISA (**K**). n = 4 retina per group for immunohistochemistry, qPCR, western blot and ELISA; repeated twice. Arrowheads in **A**–**H** indicate IL-1β and IL6-positive cells. **p < 0.01; ***p < 0.001 compared to WT group. Data represent mean ± SD. Scale bar, 50 µm.

One of the key factors in the pathogenesis of DR is the low-grade chronic inflammation driven by pro-inflammatory mediators including IL-1β and IL-6, which are thought to be released by activated macrophages and glia cells in the retina^35^. In both WT (Fig. 6A) and Akita (Fig. 6C) mice, specks of IL-1β expression were seen in the GCL, inner nuclear layer (INL) and plexiform layers. In Kimba (Fig. 6B), high expression of IL-1β was observed only in the GCL and minor staining was seen in the INL or plexiform layers. In Akimba (Fig. 6D), extensive damage was seen in all the retinal layers. In particular, the GCL was heavily stained. The INL and ONL have merged, and the entire area showed IL-1β expression. As shown in Fig. 6I, the relative mRNA expression of IL-1β increased by more than 4-fold in Akimba mouse (p < 0.001) compared with the age-matched Kimba or Akita. This increase was very well represented in the western blot (Fig. 6J), where IL-1β levels were significantly higher (p < 0.01) compared to the WT in the Akimba retina. This is further supported by the ELISA, which showed similar increases in IL-1β concentrations (p < 0.01) in the Akimba retina (Fig. 6K). Kimba or Akita retina showed an increasing trend but was not significant compared to the WT controls (Fig. 6I, J). On the contrary, we observed no increase in the IL-1α mRNA expression between WT and Akimba retina (Fig. 6I).

The WT-naïve retina (Fig. 6E) showed IL-6 immunoreactivity specifically in the nerve fiber layer (NFL) and specks of staining were also seen in the inner plexiform layer (IPL). A similar staining pattern was seen in the Akita (Fig. 6G). Kimba (Fig. 6F) showed similar staining to Akita, and few IL-6 positive cells were seen in the subretinal space. In Akimba mouse retina (Fig. 6H), IL-6 expression in NFL was even higher compared to its parental strains. More specks of staining were seen in the IPL. In addition, OPL and IS were also heavily stained. Quantitative IL-6 transcript levels followed the immunostaining trend and showed significantly higher expression (p < 0.001) exclusively in the Akimba mouse (Fig. 6I).

### NLRP3 inflammasome was activated in Akimba mouse retina

**Figure 7:**
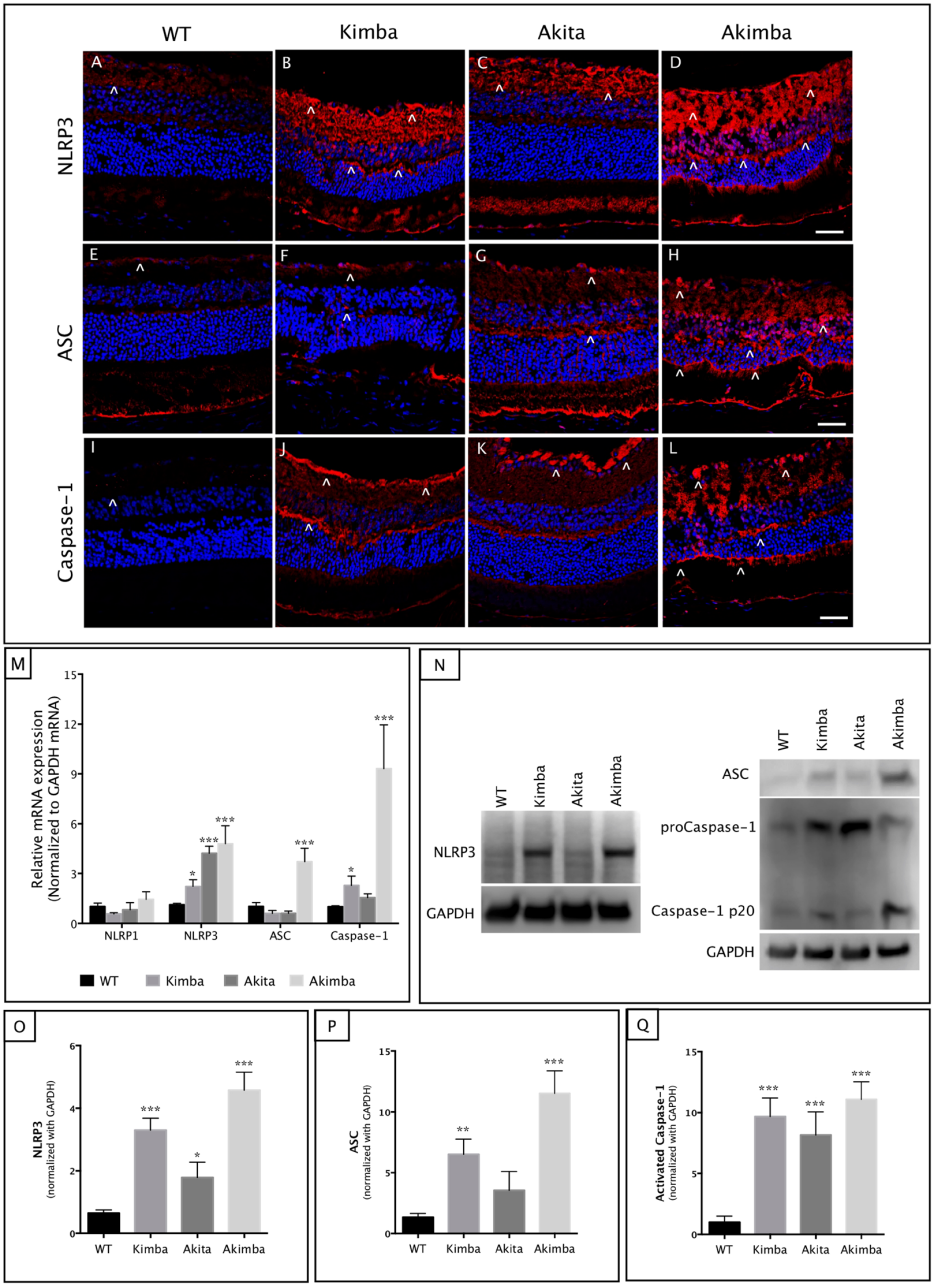
Activated retinal NLRP3 inflammasome components in Akimba mice. Retinal sections were immunostained with NLRP3 (**A**–**D**), ASC (**E**–**H**) and Caspase-1 (**I**–**L**) antibodies. Full-length blots for NLRP3 and ASC are provided in supplementary figure 1. NLRP3 was constitutively expressed in the GCL and inner plexiform layer (IPL) in the WT retina (**A**). A gradual increase in NLRP3 immunofluorescence was seen from Akita (**C**), Kimba (**B**) to Akimba (**D**). Some staining was also seen in the outer plexiform layer (OPL) indicative of the highest level of inflammatory status in the Akimba retina. Similarly, ASC and Caspase-1 were localized in the GCL and IPL in all mice models (**E**–**H** and **I–L**, respectively). ASC was also observed in the outer retina with vessel intrusion in Akimba (**H**). Caspase-1 was also seen in Akimba subretinal regions (**L**) where retinal hemorrhage was observed. Expression levels of NLRP3 components were further confirmed with real-time PCR (**M**) and western blots (**N–Q**). The most significant increase of NLRP3, ASC and Caspase-1 were seen in the Akimba compared to Kimba or Akita or WT mice. NLRP1 mRNA expression showed no differences in any of the model studied compared to the WT expression (**M**). n = 4 retina per group for immunohistochemistry, qPCR and western blot. Arrowheads in **A–L** indicate stained cells. Scale bar, 50 µm. *p < 0.05; **p < 0.01 and ***p < 0.001 compared to WT. Data represents mean ± SD.

Immunohistochemistry findings in the WT (Fig. 7A) revealed few NLRP3 positive cells in the GCL, and NFL layers. Akita showed similar staining pattern (Fig. 7C). Kimba (Fig. 7B) and Akimba (Fig. 7D) showed similar staining to Akita except that the OP was much more heavily stained and few specks in Akimba outer retina. With regard to ASC immunoreactivity, WT retinas showed expression mostly in the GCL (Fig. 7E). In Akita, the diffused staining of ASC was present throughout the retinal layers (Fig. 7G). In Kimba, there was slight staining in the GCL (Fig. 7F). Akimba showed significantly high diffused staining in the entire retina, mostly seen around the damaged retinal layers (Fig. 7H). WT retinas stained with Caspase-1 antibody exhibit diffused staining in the IPL (Fig. 7I). In Akita, NFL was heavily stained including the plexiform layers (Fig. 7K). In Kimba, the GCL and OPL were stained heavily (Fig. 7J). In Akimba, the staining was similar to Kimba but heavily stained cells were found in the outer retina (Fig. 7L).

The NLRP3 transcript levels measured using qPCR indicated progressive increase in all the animal models compared to their age-matched controls (Fig. 7M). Akimba retina showed maximal levels (~6-fold, p < 0.001) followed by Akita (p < 0.001) and Kimba (p < 0.05). In contrast, we found no differences in the NLRP1 mRNA in any of the animal model studied indicating the specificity of NLRP3 activation in these mice models. Also, we did not find any increase in inflammasome components such as ASC in Kimba or Akita retinas and their transcript levels were similar to WT controls (Fig. 7M). We found a significant increase (p < 0.05) in Caspase-1 mRNA in Kimba mice but not in Akita compared to the WT mice. On the contrary, Akimba mouse retina showed a 4-fold increase (p < 0.001) in ASC and ~12-fold increase (p < 0.001) in the Caspase-1 mRNA levels (Fig. 7M). These results corroborate with increased levels of IL-1β found only in the Akimba retina but not in Akita or Kimba mice (Fig. 6I–K). Western blots validated our findings from qPCR and immunohistochemistry (Fig. 7N–Q). Akimba retina showed a 5-fold increase in the NLRP3 (p < 0.001, Fig. 7O), a 13-fold increase in ASC expression (p < 0.001, Fig. 7P) and a 12-fold increase in the Caspase-1 (p < 0.001, Fig. 7Q) levels. Similarly, Akita showed increased NLRP3 (p < 0.05) and Caspase-1 (p < 0.001), but no significant change in ASC, while Kimba showed significantly higher levels of NLRP3 (p < 0.001), ASC (p < 0.01) and caspase-1 (p < 0.001).

### Akimba mouse retina shows increased angiogenic mediators

**Figure 8:**
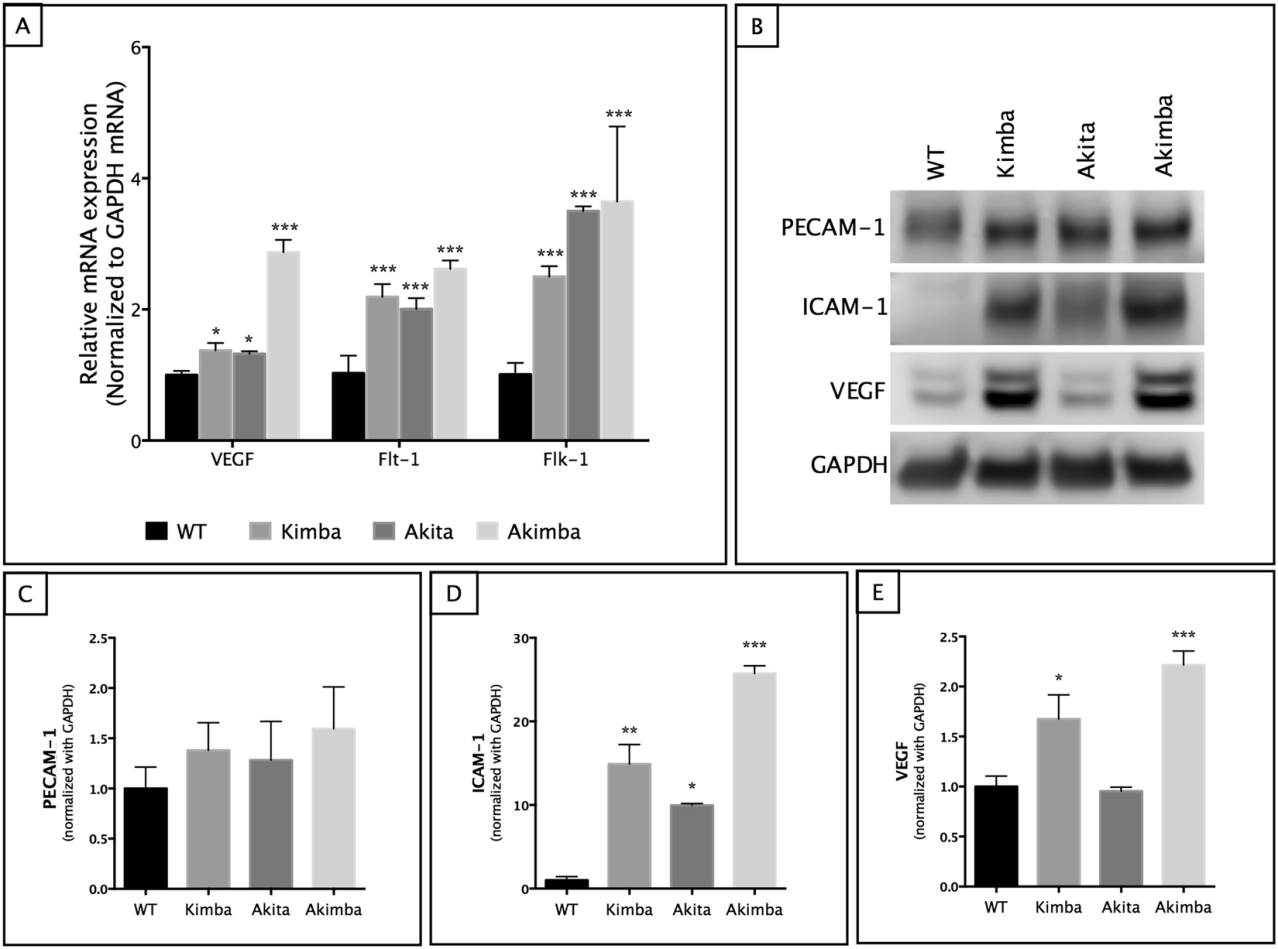
Pro-angiogenic factors were upregulated in Akimba retina. mRNA expression of VEGF showed a 3-fold increase in Akimba mouse (**A**), corresponding with a >2-fold increase in protein expression seen in Western blot (**B**,** E**). Similarly, ICAM-1 was elevated (**B**,** D**) up to 15-fold in Kimba and 25-fold in Akimba mice. PECAM-1 showed an upward trend but was not statistically significant in any of the mouse models studied (**B**,** C**). n = 4 retina per group for qPCR and western blot; repeated twice. *p < 0.05; **p < 0.01 and ***p < 0.001 compared to WT. Data represent mean $\pm$ SD.

Since PDR involves neovascularization as one of the key components in the disease pathogenesis, we studied the angiogenic mediators involved in the Akita, Kimba and Akimba mice models. We first looked at the retinal VEGF and its receptors, Flt-1 and Flk-1 that are described as the major vascular permeability factors to cause vascular leakage and pathological neovascularization^36^. We found significantly increased transcript levels of VEGF, Flt-1 and Flk-1 in all the models studied with maximal expression seen in Akimba mouse retina (Fig. 8A). Western blots showed similar results with maximum expression of VEGF detected in Akimba retina except in Akita where we found no change in VEGF protein levels compared to the WT controls (Fig. 8B–E). These results validate our earlier findings with loss of vascular permeability in Kimba and Akimba mice models but not in Akita retina (Figs 1 and 5).

We also studied other angiogenic mediators such as platelet endothelial cell adhesion molecular (PECAM)-1 and intracellular adhesion molecule (ICAM)-1, both known to play a critical role in pathological retinal neovascularization. ICAM-1 protein concentrations were significantly increased in neural retina in all animal models studied compared to the WT controls (Fig. 8D). In contrast, we found an increasing trend in PECAM-1 expression (Fig. 8C) but was not significant.

## Discussion

The inflammatory milieu in the advanced stages of DR results in the vascular leakage in DME and vaso-occlusion and retinal neovascularization in PDR^37,38^. The most challenging aspects were to find an appropriate animal model to recapitulate the cellular and pathological changes in the progression of advanced stages of human DR. Recently a double transgenic Akimba mouse model to study the advanced stages of DR has been characterized. Akimba mouse exhibit progressive retinopathic changes characterized by the photoreceptor cell death, thinning of the retina, BRB loss, vessel constriction, neovascularization, fibrosis, and edema. With aging, these mice show many clinical signs of DR including microaneurysms, retinal neuronal loss, leaky capillaries, venous beading, tortuous vessels, capillary dropout, and neovascularization. These findings suggest that Akimba mouse model represent several characteristic features of the advanced stages of human DR^29,30^. On the contrary, Kimba mouse shows several of these vascular changes but lacks diabetic background. It is noteworthy that the neovascular changes observed in the Akimba retina are not due to long-term hyperglycemia, as seen in human DR, but are due to the presence of the human VEGF_165_ transgene in the photoreceptors. Nevertheless, improvements in DR are mostly associated with the hyperglycemic control in patients only during the early stages of disease^39,40^. This is due to the fact that chronic hyperglycemic insult (>8–10 years in the human patients) causes irreversible neuronal and vascular damage in the retina, which cannot be improved merely by regulating hyperglycemia in the advanced stages of the DR.

Retinal glial cells have been implicated in the pathogenesis of DR^41^. In the early stages of diabetic retinopathy, Müller glial cells become hyperactive by releasing angiogenic and neurotrophic factors to protect the retina from hyperglycemia-induced stress. Over time, the chronic hyperglycemic insult provokes massive inflammatory milieu followed by the onset of overt neovascularization seen in the late stages of DR^41^. We found increased GFAP and GS immunoreactivity across all the animal models with maximal increase in Akimba retina accompanied by severe loss of retinal architecture and Müller cell gliosis. This was supported by the ERG data showing loss of retinal function observed in Akimba mouse. In fact, retinal neuron-to-glia cell communications are necessary intermediaries for the maintenance of blood flow dynamics^42^. This is controlled by glial cell-mediated cross-talk between neurons and endothelial cells, also referred to as neurovascular coupling. The recent discovery of a novel laser speckle flowgraphy (LSFG) imaging technique for the evaluation of the microvasculature had led to the development of a rapid and non-invasive method for the determination of basal blood flow and neuronal activity-dependent alterations in ocular diseases^32^. LSFG has been suggested to serve as a useful biomarker not only for detection but also for monitoring postoperative visual outcomes in the patients with retinal and choroidal vascular diseases^43,44^. In type 1 and type 2 diabetic patients, a substantial decrease in the flicker-evoked vasodilation was observed^45–47^, which suggests reduced neurovascular coupling causing hypoxia and eventually retinal neovascularization. We found a significantly reduced blood flow volume in the Akimba retina compared to their parental strains depicting a dysregulated retinal microvasculature.

Next, we looked at the resident and migratory retinal microglial cells using multiple rodent microglia/macrophage cell markers. Isolectin-B4-stained Akimba retinal flat mounts showed a significantly high degree of leaky and degenerated retinal vasculature compared to the WT or parental strains. Whether or not the retina contains antigen-presenting dendritic cells has been a topic of controversy for decades. Zhang and colleagues reported a small population of MHC-II^+^ cells in the rat retina^48^. A recent study by Rakoczy and group showed a dramatic increase in MHC class II^+^ cells throughout the retina in Akimba mice^29^. In the normal retina, microglial cells are long slender processes distributed in the inner and outer plexiform layers (IPL & OPL), GCL and nerve fiber layer (NFL), showing highly motile and ramified protrusions. In DR, the microglia increase in number and become hypertrophic (amoeboid) with shorter dendrites and larger somas at different stages of DR. Often, they resemble perivascular macrophages in the area of retinal injury and cluster around the vasculature^49^. We used Iba-1 and OX-42 as well as F4/80 and CD14 double immunostaining to distinguish the resident or migratory microglia and perivascular macrophages in the retina. Although both perivascular macrophages and microglia express Iba-1, OX-42 and F4/80, CD14 is highly expressed by infiltrating macrophages and not by microglial cells^10,49^. We found a progressive increase in the Iba-1 expressing cells co-expressed with OX-42 in the WT to Akita to Kimba retina to maximal expression was observed in Akimba, where Iba-1/OX-42 positive cells were seen in the outer retina. This could be due to the loss of BRB in the Akimba mouse. The expression of F4/80 in WT was meager but found upregulated in the inner retinal layers of the Akita mouse presumably because of the damaged inner BRB (iBRB)^18^. In Akimba mice retina, significantly increased F4/80 and CD14 positive cells were detected in the outer retinal layers which could be either due to the migration of activated resident macrophages or from the infiltration of activated circulating macrophages from the choroid into the retina through the compromised oBRB. It is noteworthy that we observed only a few cells co-stained with CD14 and F4/80, which could be due to the fact that CD14 expressing perivascular macrophages might have migrated to the outer retina^50^ and represent a different subset of retinal macrophages as reported earlier^10^. In addition, in DME, microglia can escape outer limiting membrane and migrate to the outer retina and subretinal space. In fact, the activation and proliferation of microglia have been reported in both the inner and outer retina in the Goto-Kakizaki rat model (GK) of DR^51^. However, the relative importance and the role of resident and migrating microglia/macrophages in the pathogenesis of DR is yet to be fully determined.

During the advanced stages of DR, chronic neuroinflammation released several pro-inflammatory mediators and increased oxidative and nitrosative stress that may compromise the BRB and contribute towards neuronal and vascular damage in the retina. Increased levels of IL-1β and IL-6 have been found in the serum and vitreous of patients with DR^8,9^. Uncontrolled release of IL-1β has also been suggested to play a crucial role in the formation of acellular capillaries and retinal capillary cell death^35^. Consistent with the previous report, we found a significant increase in IL-1β and IL-6 expression in the inner retinal layers of Akita including the plexiform layers, GCL and NFL. The most deleterious effects were seen in the Akimba neural retina with significantly high levels of IL-6 and IL-1β transcripts and protein levels. Indeed, a previous study has shown IL-1β expression in microglial and macroglial cells (astrocytes and Müller cells) localized adjacent to RGCs^52^. Hence, we propose that microglial cells are the major producers of secreted IL-1β and IL-6 further elevating an inflammatory response supported by a previous finding that knocking out IL-1β receptor protected the retinal capillaries from degeneration in diabetic animals^53^.

Since IL-1β can amplify the initial inflammatory response by activating Caspase-1 in an autocrine manner, we next looked at the regulation of IL-1β activation in our transgenic mouse models. It has been recently reported that both IL-1β maturation and its release are tightly regulated by NLRP3 inflammasome, which has been involved in several systemic inflammatory diseases such as gout, atherosclerosis, pulmonary fibrosis and contact hypersensitivity^54^. In addition, NLRP3 inflammasome has been reported to be a critical component in the pathogenesis of metabolic disorders such as obesity and type 2 diabetes. Very recently, a clinical study suggested the association of inflammasome in the vitreous of the PDR patients^22^. The NLRP3 inflammasome is best characterized as a protein scaffold upon which an ASC adaptor assembles and acts to recruit pro-Caspase-1.

Clinical studies have demonstrated NLRP3 inflammasome-mediated release of IL-1β in diabetic conditions can be therapeutically treated with IL-1β antagonist^55,56^. In addition, mice deficient in the components of NLRP3 have been shown to have reduced diabetic symptoms. A previous study has reported an increase in Caspase-1 activity in retinas of diabetic mice, galactose-fed mice, diabetic humans, and in retinal Müller cells treated with high glucose^57^. In the present study, NLRP3 was upregulated in the inner retina along with Caspase-1 and ASC expression compared to the WT retina. Although we found increased transcripts of NLRP3 components in all the animal models studied significantly higher levels of IL-1β protein were exclusively seen in the Akimba mouse retina using western blot and ELISA. On the contrary, NLRP1 inflammasome remained unchanged in any of the models studied, thus indicating the specificity of NLRP3 inflammasome activation in the retina. This suggests that NLRP3 inflammasome plays a key role in the Akimba retina to release pro-inflammatory mediators eliciting a prolonged inflammatory response involved in the pathogenesis of DR. However, the *in vivo* functional role of NLRP3 in PDR needs further studies.

Another predominant feature seen in late stages of DR is retinal neovascularization caused by increased vascular permeability. This leads to VEGF upregulation and activates Flk-1 on endothelial cells (ECs) to promote the aberrant formation of new vessels^18^. In addition, the VEGF/Flk-1 signaling axis facilitates the degradation of adhesion junctions and loosens the intercellular junctions between neighboring ECs^58^. We found a significant increase in the VEGF levels in Akimba retina (and a mild increase in Kimba) contrary to the increased VEGF, or its receptor transcripts in all the mice models studied. A previous study suggests that VEGF injected in naïve eyes triggered many of these vascular changes observed during diabetes, such as ICAM-1 upregulation and vascular permeability^59^. Similarly, we found an increased expression of intercellular adhesion molecule (ICAM)-1 in the retina at transcript levels as well as in protein levels in Akimba mice.

In summary, our study suggests that the dysregulated activation of the NLRP3 inflammasome in the Akimba mouse compromises retinal immune response resulting in the loss of BRB. These changes led micro- and macroglia cells to a continuous activated state causing vascular leakage and retinal neovascularization observed in the advanced stages of DR.

## Materials and Methods

### Animals

Akita, Kimba, and Akimba mice were procured from Centre of Ophthalmology and Visual Science, The University of Western Australia, Crawley, Australia and inbred for at least 10 generations. Akita mice have a dominant point mutation in the insulin-2 gene, making it a natural occurring type-I diabetic mouse model. Kimba mice models transiently overexpress human VEGF165 in the photoreceptors, hence depicts advanced stage neovascularization in the retina. The Akimba mice developed by Rakoczy *et al.*^29^ were generated by crossing homozygous Kimba with heterozygous Akita mice. All mice are on C57BL/6 J background and inbred for at least 10 generations before used for experiments. Six-months-old mice (20–24 weeks) were used in this study. There were a minimum of 6 animals in each experiment unless otherwise stated in the figure legends. Animals were housed in 12 h light/12 h dark cycle with food and water provided ad libitum.

### Ethics Statement

All mice experimental procedures was approved by the Singhealth Institutional Animal Care and Use Committee (IACUC), Singapore and were conducted in accordance with the ARVO recommendations for animal experimentation, and data presented in accordance with the ARRIVE guidelines.

### Fundus and Fluorescein Angiography (FFA)

Digital color fundus photographs were taken using MICRON IV (Phoenix Research Laboratories, Pleasanton, CA, USA). Mice were anesthetized with a combination of 20 mg/kg ketamine hydrochloride (Ketamine, Parnell Laboratories, NSW, Australia) and 2 mg/kg xylazine hydrochloride (Ilium Xylazil-20, Troy Laboratories, NSW, Australia). The eyes were dilated with 1% tropicamide (Alcon Laboratories, Inc., Fort Worth, TX, USA), followed by 2.5% phenylephrine (Bausch and Lomb Pharmaceuticals, Inc., Tampa, FL, USA). A small drop of coupling gel (Genteal, Novartis Pharmaceuticals, East Hanover, NJ, USA) was placed on each eye to prevent corneal dryness. For FFA, mice were intraperitoneally injected with 10% sodium fluorescein dye (AKORN, Lake Forest, IL, USA) diluted by 10× in sterile saline, and given at a dose of 0.1 ml/10 g body weight. For accurate comparison of fluorescein intensity in the retina, FFA images were taken 30 s after injection, in the sequence as follows: central, temporal, nasal, superior, inferior. One image was taken per retinal area, to avoid fluorescein saturation. Semi-quantitative analysis of fluorescein intensity was performed by measuring the mean gray value of FFA image from each area of the retina, and values obtained from Kimba, Akita and Akimba mice were normalized to the WT mice for relative fold intensity.

### OCT

Image-guided spectral-domain OCT images were taken using inSight software (Phoenix Research Labs). Genteal was applied to the eyes before imaging to keep cornea moist. Trans-retinal images were taken from regions around the optic nerve. Retinal thickness was calculated in the software.

### Electroretinography (ERG)

Mice were dark-adapted for a minimum of 12 hours. All ERG recordings were performed using an Espion system (Diagnosys LLC, Lowell, MA, USA) in a dark room under dim red light illumination. Mice were anesthetized, and the eyes were dilated as described above. Body temperature was maintained at 37 °C using pumped-water heat pad fixed on the ERG stage. Coupling gel was applied to each eye, before placing a monopolar electrode on the cornea. A gold-cup electrode was placed in the mouth to serve as the reference electrode, and a silver-silver chloride electrode was inserted into the tail as the ground electrode. The mouse head was positioned in front of a Ganzfeld stimulus. Scotopic ERG responses were recorded across increasing light intensities from −3.3 to 1.5 log cd.s.m^−2^ in 0.3-log-unit increments. Full-field ERG was recorded, and each response was the average of 3–5 trials. Signals were acquired at 2 kHz with high- and low-pass filtering at 0.1 and 100 Hz respectively.

### Laser Speckle Flowgraphy (LSFG)

LSFG uses a laser speckle phenomenon to measure the real-time 2-dimensional fundus blood flow changes without pupillary dilations or contrast media providing new insights into the pathophysiology of retinal diseases. Movement of blood cells in the tissue disrupts the speckle pattern of laser irradiation on the retina, which can be expressed as flow rate. Mice were anesthetized, and eyes dilated as described in FFA section. They were placed into the stand of the LSFG with one eye facing downward. Coupling gel was applied to the top eye before a cover glass was placed onto the cornea. LSFG (Softcare Co. Ltd., Fukuoka, Japan) was performed at the optic nerve head of the eye. Images were acquired continuously for 4 s to produce a composite map of ocular blood flow. The procedure was repeated with the contralateral eye. Calculation of retinal flow volume was produced by first subtracting the background choroidal flow from the overall blood flow value of a region of interest centered on a retinal vessel, reflecting retinal flow velocity and vascular diameter. Up to 6 areas of a blood flow map were analyzed. All calculations and analysis were performed by a single masked observer.

### Tissue Harvesting and Analysis

For analysis by real-time PCR, ELISA and western blot, enucleated eyes were dissected immediately on chilled PBS and processed as described previously^60^. For immunohistochemistry, enucleated eyes were first fixed in 4% paraformaldehyde (PFA) in 1× PBS. Eyes were treated to sucrose gradient (15% and 30%) cryoprotection before embedding in Tissue Freezing Medium® (Leica Biosystems Inc., Buffalo Grove, IL, USA) on dry ice. Retina sections of 6–8 µm thickness were cut using HM525 HX Cryostat (Thermo Fisher Scientific, Waltham, MA, USA) and collected on Polysine™-coated glass slides (Thermo Fisher Scientific). Glass slides were kept frozen at −80 °C until staining was performed. For flat mount staining, eyes fixed in 4% PFA for 10 min was hemisected ora ciliaris to isolate the neural retina. Flat mount staining was performed immediately.

### Real-Time PCR

Neural retina tissue was homogenized using a handheld homogenizer (Sigma-Aldrich, St. Louis, MO, USA) in RLT buffer +1% β-mercaptoethanol. RNA was isolated and purified using RNeasy Mini Kit (Qiagen, Valencia, CA, USA) according to manufacturer’s instructions. cDNA was generated using GoScript reverse transcriptase (Promega, Madison, WI, USA). 250 ng of RNA was used as the starting materials. Real-time PCR with SYBR Green dye (Thermo Fisher Scientific) was conducted on the LightCycler 480 II System (Roche, Indianapolis, IN). The PCR run starts with preincubation at 95 °C for 5 min, followed by 45 amplification cycles at 95 °C for 10 s, 60 °C for 15 s, and 72 °C for 30 s. Melt curve analysis was conducted to verify amplification reaction. GAPDH was used as housekeeping gene. Primer sequences are listed in Supplementary Table 1.

### Immunohistochemistry

Immunofluorescence staining was performed according to standardized protocol described earlier^60^. In brief, frozen slides were kept at room temperature for 15 min, then soaked in 1× PBS for 10 min. Tissue sections were post-fixed with 4% PFA for 10 minutes followed by two washes in 1× PBS for 5 min each. Blocking was done with 1% BSA for 20 minutes at room temperature. Primary antibody was incubated overnight at 4 °C, and secondary antibody incubated for 1 h at room temperature the following day. Double immunostaining was performed by sequential overnight incubations with antibodies directed for two different species. Slides were mounted with Vectashield® Mounting Medium with DAPI (Vector Laboratories, Burlingame, CA, USA). Retinal sections without primary antibody served as staining control. The list of antibodies used in this study is listed in Supplementary Table 2.

For flat mount with isolectin GS-IB4, Alexa Fluor® 594 conjugate (Thermo Fisher Scientific), flat mounts were washed in 1× PBS, blocked with PBS + 1% Triton X-100 (PBSTx), and left overnight with 1:100 antibody dilution in PBSTx. Flat mounts were cut into four quadrants and mounted with ProLong® Diamond antifade mountant (Thermo Fisher Scientific). Slides were kept at 4 °C for at least 12 hours before viewing. All sections were viewed and imaged on the Confocal Micros C2 (Nikon Instruments Inc, Melville, NY, USA).

### Western Blotting

Total protein from each neural retina was extracted in 150 µl of RIPA buffer + protease and phosphatase inhibitor (Sigma-Aldrich) using handheld homogenizer. Protein quantification was performed using BCA protein assay kit (Pierce, Thermo Fisher Scientific). 15 µg of protein was loaded onto 4–20% pre-cast gels (Bio-Rad, Hercules, CA, USA). Electrophoresis was run at 200 V for 30 min. The transfer was made onto PVDF membrane overnight at 40 V in 4 °C. Immunoblotting was performed using 5% milk. All primary antibodies were incubated overnight at 4 °C, and secondary antibody was kept for 1 h at room temperature. Protein bands were visualized using SuperSignal Chemiluminescence (Pierce). Antibodies are listed in Supplementary Table 2. GAPDH was used as loading control.

### ELISA

To further validate the expression of IL-1β, total expressed and secreted IL-1β was measured using mouse-specific IL-1 beta Quantikine ELISA kit (R&D Systems, Minneapolis, MN, USA) according to manufacturer’s instructions. Retina protein lysate was taken from homogenate made for western blot. Colorimetric readings were taken at 540 nm. The correction was made by subtracting readings from 450 nm.

### Statistical Analysis

Statistical analysis was performed using Student t-test for comparison between 2 groups (GraphPad Prism 6.0, GraphPad Software, Inc., La Jolla, CA, USA). Comparison between multiple groups was done using one-way ANOVA followed by post-hoc analysis using Newman-Keuls test. Significance was taken when p < 0.05. Data were presented as mean ± SD. The number of animals or tissues used per analysis and repeats of each experiment are described in the figure legends.

## Electronic supplementary material

Supplementary Figure 1
