# Supplementary material for: The NLRP3 Inflammasome May Contribute to Pathologic Neovascularization in the Advanced Stages of Diabetic Retinopathy
Source: Sci Rep. 2018 Feb 12;8:2847. doi: 10.1038/s41598-018-21198-z (PMC5809448; doi:10.1038/s41598-018-21198-z)
Supplement: Supplementary file 1 — Supplementary Figure 1 [file 41598_2018_21198_MOESM1_ESM.pdf]

The NLRP3 Inflammasome May Contribute to Pathologic Neovascularization in the Advanced Stages of Diabetic Retinopathy

Shyam S Chaurasia[1,2,3,*ξ], Rayne R. Lim[1,2,3], Bhav H. Parikh[4], Yeo Sia Wey[4], Bo Bo Tun[4], Tien Yin Wong[4,5], Chi D Luu[6], Rupesh Agrawal[7], Arkasubhra Ghosh[8], Alessandra Mortellaro[9], Elizabeth Rackoczy[10], Rajiv R. Mohan[1,2,3,11], Veluchamy A. Barathi[4,5,ξ]

[1]Ocular Immunology and Angiogenesis Lab, Department of Veterinary Medicine & Surgery, University of Missouri, Columbia, MO, USA
[2]Department of Biomedical Sciences, University of Missouri, Columbia, MO, USA
[3]Ophthalmology, Harry S. Truman Memorial Veterans' Hospital, Columbia, MO, USA
[4]Translational Pre-Clinical Model Platform, Singapore Eye Research Institute, Singapore
[5]The Ophthalmology & Visual Sciences Academic Clinical Program, DUKE-NUS Graduate Medical School, Singapore
[6]Centre for Eye Research Australia, Department of Surgery (Ophthalmology) University of Melbourne, Melbourne, Australia
[7]National Healthcare Group Eye Institute, Tan Tock Seng Hospital, Singapore.
[8]GROW laboratory, Narayana Nethralaya, Bangalore, India
[9]Singapore Immunology Network (SIgN), Agency for Science, Technology and Research (A*STAR), Singapore
[10]Centre for Ophthalmology and Visual Sciences, University of Western Australia, Perth, WA 6009, Australia
[11]Mason Eye Institute, University of Missouri, Columbia, MO, USA

***Corresponding author:**

Shyam S. Chaurasia, MS, PhD
Assistant Professor of Ophthalmology and Vision Sciences
Ocular Immunology and Angiogenesis Lab
One Health One Medicine Ophthalmology Research Program
Department of Veterinary Surgery & Medicine
University of Missouri
Rm 115A, 1600 E Rollins Street
Columbia, MO 65211
Phone: 573-639-7033

Legends to Supplementary Figure

Supplementary Figure 1: The full-length version of the western blots of wild-type (WT), Kimba, Akita and Akimba retinal samples probed with (A) Rabbit mAb (mouse specific) NLRP3 and (B) Rabbit polyclonal ASC Ab. The predicted size for NLRP3 was 110 kDa, and ASC was 24 kDa.

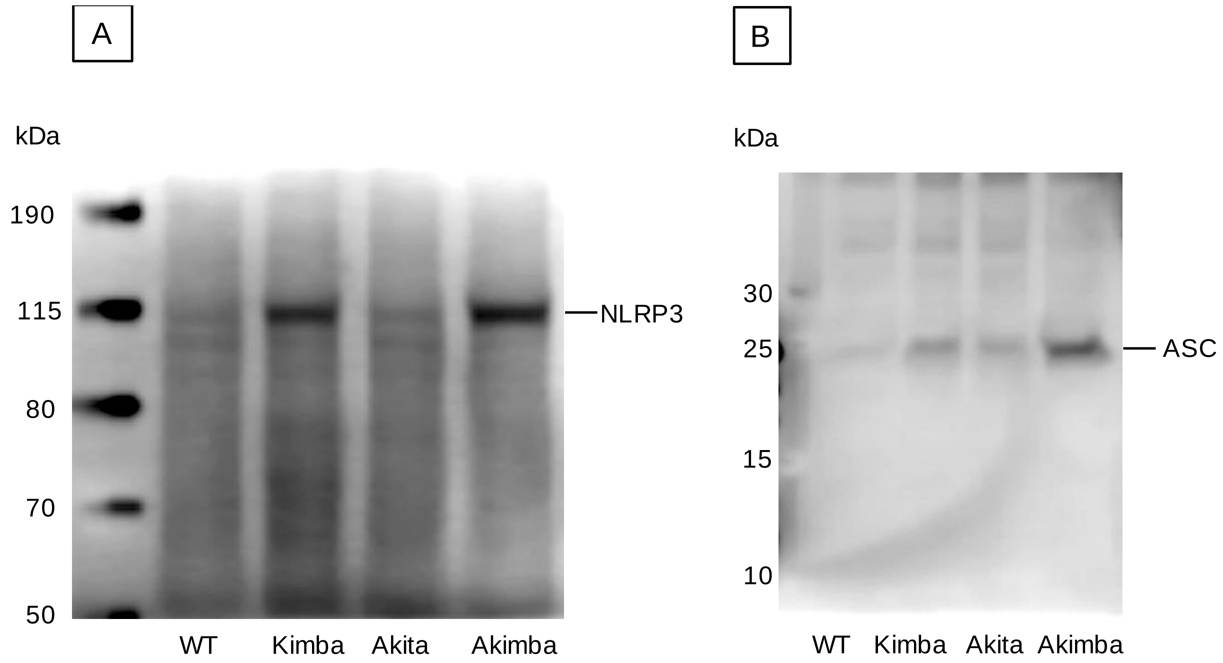

Legends to Supplementary tables

Supplementary Table 1. Primer sequences used in the real time PCR.

Gene	Accession		Sequence (5'-3')	Amplicon
**NLRP1**	NM_001004142.2	Forward	ACCTTCCAAATCACTTCCTCTT	139
		Reverse	GTCTGATTCCAGGTCTCCATTT	
**NLRP3**	NM_145827.3	Forward	CTTCTAGCTTCTGCCGTGGTCTCT	189
		Reverse	CGAAGCAGCATTGATGGGACA	
**Caspase-1**	NM_009807.2	Forward	GTACACGTCTTGCCCTCATTATCTG	167
		Reverse	TTTCACCTCTTTCACCATCTCCAG	
**ASC**	NM_023258.4	Forward	CTGAGCAGCTGCAAACGACTAAA	135
		Reverse	CTTCTGTGACCCTGGCAATGAGT	
**IL-1α**	NM_010554.4	Forward	GGAGAAGACCAGCCCGTGTTGCT	180
		Reverse	CCGTGCCAGGTGCACCCGACTT	
**IL-1β**	NM_008361.3	Forward	CAACCAACAAGTGATATTCTCCATG	152
		Reverse	GATCCACACTCTCCAGCTGCA	
**VEGF**	NM_001025250.3	Forward	CACAGCAGATGTGAATGCAG	186
		Reverse	TTTACACGTCTGCGGATCTT	
**Flt-1**	NM_010228.3	Forward	GAGGAGGATGAGGGTGTCTAT	105
		Reverse	CAGGTTTGACTTGTCTGAGGT	
**Flk-1**	NM_010612.2	Forward	AGAGCGATGTGTGGTCTTTC	126
		Reverse	CCCGCATTCTAGTTCCTTCTT	
**GAPDH**	NM_008084.3	Forward	CATGGCCTCCAAGGAGTAAGA	105
		Reverse	GAGGGAGATGCTCAGTGTTGG	

Supplemenatry Table 2. List of primary antibodies used in the study.

Antibody	Company	Catalogue No.
**Iba-1**	Wako	019-1971
**OX-42**	BD Pharmingen	550282
**F4/80**	Abcam	ab6640
**CD14**	Abcam	ab203294
**GFAP**	Dako	Z0334
**GS**	Sigma Aldrich	G2781
**NLRP3**	Cell Signaling	15101
**NLRP3**	Santa Cruz	sc-66846
**ASC**	Adipogen	AG-25B-0006
**Caspase-1**	Santa Cruz	sc-514
**Caspase-1**	Adipogen	AG-20B-0042
**IL-1β**	Santa Cruz	sc-7884
**IL-6**	Cell Signaling	12912
**PECAM-1**	Cell Signaling	77699S
**ICAM-1**	Santa Cruz	sc-107
**VEGF**	Abcam	ab46154
**GAPDH**	Santa Cruz	sc-25778
